# Clathrin mediated endocytosis in Alzheimer’s disease: cell type specific involvement in amyloid beta pathology

**DOI:** 10.3389/fnagi.2024.1378576

**Published:** 2024-04-17

**Authors:** Sierra Jaye, Ursula S. Sandau, Julie A. Saugstad

**Affiliations:** Department of Anesthesiology & Perioperative Medicine, Oregon Health & Science University, Portland, OR, United States

**Keywords:** Alzheimer’s disease, amyloid beta, clathrin mediated endocytosis, endolysosomal dysfunction, therapeutics

## Abstract

This review provides a comprehensive examination of the role of clathrin-mediated endocytosis (CME) in Alzheimer’s disease (AD) pathogenesis, emphasizing its impact across various cellular contexts beyond neuronal dysfunction. In neurons, dysregulated CME contributes to synaptic dysfunction, amyloid beta (Aβ) processing, and Tau pathology, highlighting its involvement in early AD pathogenesis. Furthermore, CME alterations extend to non-neuronal cell types, including astrocytes and microglia, which play crucial roles in Aβ clearance and neuroinflammation. Dysregulated CME in these cells underscores its broader implications in AD pathophysiology. Despite significant progress, further research is needed to elucidate the precise mechanisms underlying CME dysregulation in AD and its therapeutic implications. Overall, understanding the complex interplay between CME and AD across diverse cell types holds promise for identifying novel therapeutic targets and interventions.

## 1 Introduction

**Figure 1:**
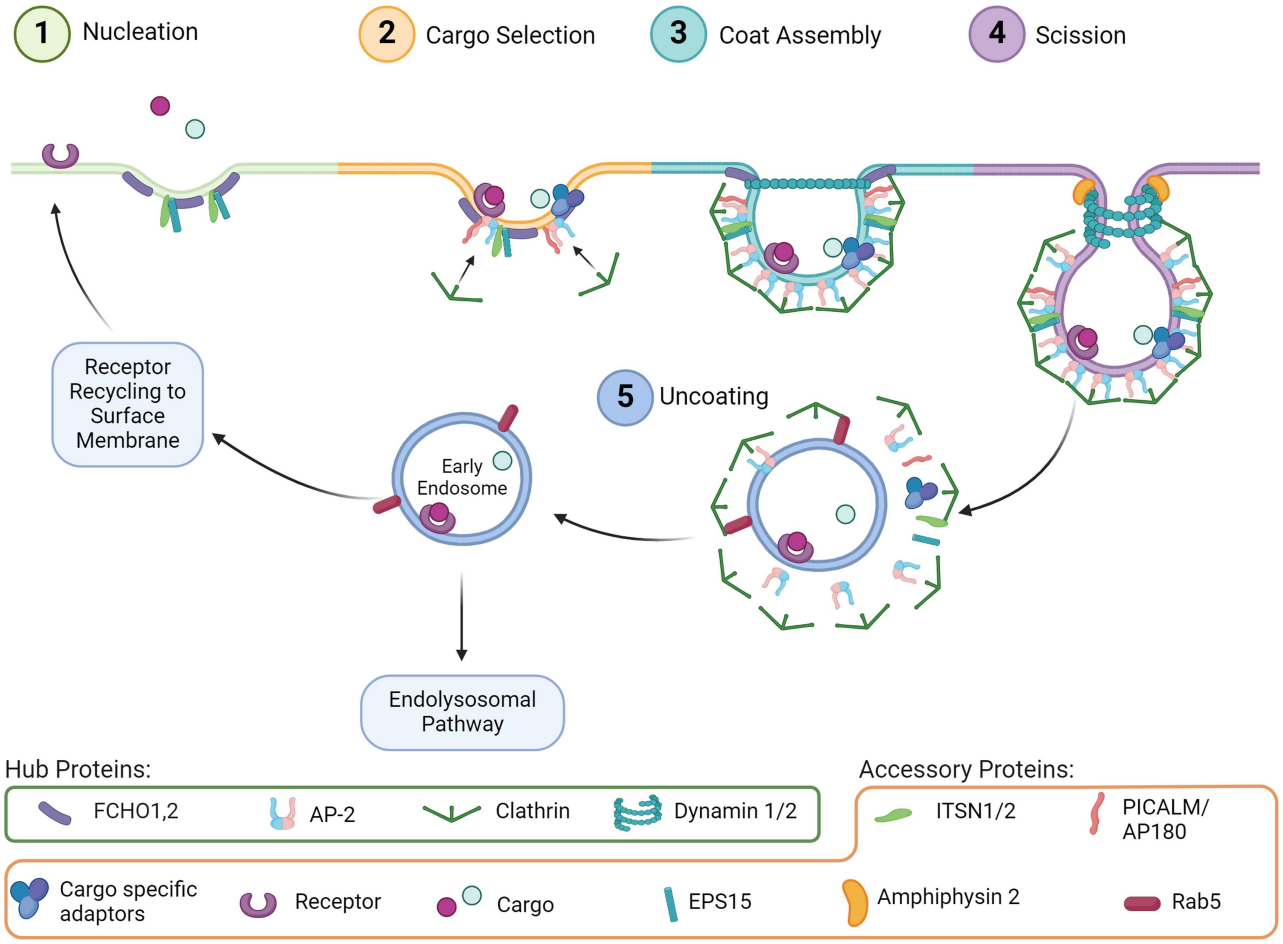
CME step-by-step. (1) Nucleation: Initial invagination of the membrane surface and recruitment of support proteins to the pit. (2) Cargo selection: Budding vesicle couples with the intended cargo via receptors and adaptor proteins, which concurrently begin recruiting clathrin. (3) Coat assembly: Clathrin begins forming a lattice around the budding vesicle. (4) Scission: Dynamin assembles around the vesicle neck, and with support from accessory proteins the vesicle is removed from the extracellular membrane. (5) Uncoating: The clathrin lattice disassembles and the early endosome can be trafficked through several pathways including back to the cell surface or through the endolysosomal pathway.

Endocytosis is a ubiquitous and essential function of mammalian cells as it mediates many downstream pathways through the internalization of extracellular material from the cell surface. Many types of endocytosis have been described, including clathrin-mediated endocytosis (CME), dynamin-dependent endocytosis, caveolin dependent endocytosis, micropinocytosis, and phagocytosis (Doherty and McMahon, 2009). Of these, CME is one of the most well characterized and mechanistically understood. Processes that rely on CME include uptake of intracellular signals, transmembrane receptor recycling, regulation of membrane composition, synaptic vesicle recycling, and initiation/regulation of downstream intercellular signaling cascades such as the endolysosomal system. The distinct phases of CME are comprised of nucleation, cargo selection, coat assembly, scission, and uncoating. Each step is moved forward by an essential hub protein, which are supported by additional accessory proteins, described in detail through decades of studies (McMahon and Boucrot, 2011; Kaksonen and Roux, 2018). Hub and accessory proteins are indicated in Figure 1.

CME is initiated by proteins involved in nucleation (Figure 1, Step 1), including FCH and mu domain containing endocytic adaptor 1/2 (FCHO1,2), Intersectin1 (ITSN1), and Epidermal growth factor receptor substrate 15 (EPS15). These proteins cluster and bind to the plasma membrane to initiate membrane curvature and recruit other proteins, such as the Adaptor protein 2 complex (AP-2), for cargo selection (Figure 1, Step 2). Clathrin is also recruited by AP-2, which then begins the clathrin coat assembly (Figure 1, Step 3) of the endosome and curves the membrane further, forming the vesicle around the clustered cargo. The vesicle then undergoes scission from the membrane (Figure 1, Step 4) by recruitment of Dynamin via the BAR-domain containing proteins. Uncoating of clathrin (Figure 1, Step 5) is subsequently initiated by Auxilin of G-associated kinase (GAK), which recruits ATPase HSC70 to disassemble the clathrin coat allowing the machinery to be available for the next endocytosis event. Cargo internalized through CME is typically sorted through the endolysosomal pathway for delivery to the appropriate cellular compartment (Boulant et al., 2011; McMahon and Boucrot, 2011; Mettlen and Danuser, 2014).

Given the fundamental need for cells to utilize this process, there are many diseases associated with disruption of CME. However, dysfunction is more common in accessory proteins, as genetic mutations of essential hub proteins, for example those that make up the AP-2 complex, can be embryonic lethal (Mitsunari et al., 2005; Azarnia et al., 2019). Changes in accessory proteins can still have profound effects on CME function and are associated with cancer, psychiatric diseases, and various neurodegenerative diseases (McMahon and Boucrot, 2011). For example, in Parkinson’s disease, mutations in synaptojanin and auxilin have been shown to be causative for early onset Parkinson’s (Schreij et al., 2016). In Alzheimer’s disease (AD), genetic links have been made to endocytic protein genes such as *AP-2* (cargo selection), Clusterin (*CLU*, cargo selection), Bridging integrator 1 (*BIN1*, scission), CD2 associated protein (*CD2AP,* scission/uncoating), and Phosphatidylinositol binding clathrin assembly protein (*PICALM*, cargo selection) (Raj et al., 2018; Azarnia et al., 2019; Ando et al., 2022; Szabo et al., 2022). *BIN1* and *PICALM* are two of the most significant risk factors for late onset AD (Andrade-Guerrero et al., 2023). Importantly, while not directly involved in CME, many other significant AD risk factors like Apolipoprotein E (*APOE*), Ras and rab interactor 1 (*RIN1*), and Sortilin related receptor 1 (*SORL1*) have been related to endosomal and lysosomal dysfunction downstream from CME and are reviewed elsewhere (Szabo et al., 2022; Maninger et al., 2024).

AD is the most common form of dementia (Alzheimer's Association, 2023) and is characterized by the progressive development of amyloid beta (Aβ) plaques and neurofibrillary Tau protein tangles in the brain (Braak and Braak, 1991; Selkoe and Hardy, 2016). AD is often diagnosed 15–20 years after pathology begins to develop, so there is a need for reliable tools to detect early brain changes that lead to it (Deture and Dickson, 2019) and the effort to identify early cellular changes before the onset of symptoms is of great importance. As AD progression is a highly complex process, many mechanisms have been associated with disease progression, such as neuroinflammation, impaired autophagy, oxidative stress, vascular dysfunction, and endolysosomal dysfunction (Nixon et al., 2000; Theerasri et al., 2022). Perhaps one of the most widely referenced mechanisms is the Aβ cascade hypothesis, a well-established model of disease progression, in which intercellular Aβ accumulates as plaques in the brain, induces Tau pathology, and ultimately neuronal death (Hardy and Allsop, 1991; Hardy and Higgins, 1992). Others have since suggested that the imbalance of extracellular Aβ clearance and intracellular accumulation of Aβ initiates neuronal dysfunction rather than merely the accumulation of extracellular plaques (Selkoe and Hardy, 2016; Volloch and Rits-Volloch, 2022, 2023). As such, the processes of Aβ production and clearance have been of particular interest in the field of AD.

The pathogenic form of Aβ (Aβ_1-42_) is produced when Amyloid precursor protein (APP) is endocytosed and processed in the endolysosomal pathway of neurons (Koo and Squazzo, 1994; Selkoe and Hardy, 2016). CME has also been related to Tau pathology, primarily as a function of intercellular propagation in which aggregated pathologic Tau (Lamontagne-Kam et al., 2023) is internalized via many types of endocytosis including CME (Ando et al., 2020). While the potential cellular effects of Tau internalization by endocytosis are substantial (Thal and Tome, 2022), the ratio of Tau internalized via CME versus other endocytic pathways is unknown and thus studies are needed to determine if CME is as prominent a mechanism of Tau internalization, as it is for Aβ uptake.

As the primary process that feeds into the endolysosome, disruption of CME has the potential to affect this and many other downstream cellular pathways implicated with AD. The endolysosomal pathway in AD has been extensively studied downstream of CME (Nixon, 2017; Szabo et al., 2022), yet few investigators have evaluated the upstream changes and contribution of actual endocytosis, specifically CME, to AD pathogenesis. Additionally, in other brain cell types like microglia and astrocytes, CME is implicated in extracellular debris uptake and could be important to Aβ_1-42_ clearance, illustrating the potential for CME to affect the balance of both intra- and extra-cellular Aβ_1-42_ accumulation in multiple ways via multiple cell types. Here, we discuss how CME in different brain cell types has thus far been implicated in AD risk and progression. Aside from neurons, there is a gap in knowledge regarding a role for CME in Tau pathology. Thus, we will be focusing on CME and its relation to Aβ but will include Tau where information is available. We also focus the review on research that examines the importance of CME as a potential early disease modifier. For a detailed review of the role of downstream endosomal trafficking on AD pathogenesis see (Nixon, 2020; Behl et al., 2022; Szabo et al., 2022).

## 2 AD and endolysosomal function

The endolysosomal pathway systematically internalizes and sorts extracellular molecules that can either be used in the cell, recycled back to the cell membrane, or degraded internally through lysosomes and autophagosomes. Classically, molecular cargo is internalized through CME or other endocytic pathways and trafficked to early endosomes and then late endosomes, recycled to the surface membrane, or sent to the lysosome for degradation (Cullen and Steinberg, 2018). In AD, defects in the endolysosomal pathway cause cellular stress in multiple ways, including decreased endosomal transport, altered endosome signaling, reduction in vesicle recycling, decreased lysosomal acidification, and oxidative stress. These defects then result in various neuropathological outcomes such as Aβ_1-42_ over production and clearance deficits, as well as dystrophic neurites (Nixon, 2017; Colacurcio et al., 2018; Szabo et al., 2022).

Interestingly, the earliest presenting cytopathology in sporadic AD, even before accumulation of extracellular Aβ plaques are observed, is enlargement of early endosomes in neurons containing soluble Aβ (Cataldo et al., 2000, 2004). Indeed, this intracellular Aβ frequently associated with the enlarged endosomes (Cataldo et al., 2004). This phenotype is specific to sporadic AD and has not been observed in familial AD or other neurodegenerative diseases like Parkinson’s and Huntington’s disease (Cataldo et al., 2000). Enlarged early endosomes are also observed in induced pluripotent stem cell (iPSC) derived neurons from sporadic AD patients (Israel et al., 2012) and iPSC lines with *APP* and presenilin 1 (*PSEN1*) mutations, both genetic risk factors for early-onset AD (Kwart et al., 2019; Lacour et al., 2019). Interestingly, endolysosomal dysregulation and enlarged early endosomes are also seen in Down syndrome, a genetic disorder caused by a triplication of chromosome HSA21 (Cataldo et al., 2000). People with Down syndrome have a significantly higher risk of developing AD, with nearly all patients developing neuropathology and symptoms by age 65 (McCarron et al., 2017; Fortea et al., 2021). This phenomenon is thought to be due to several AD related genes encoded on HSA21, such as *APP* and *PSEN1* (Colacurcio et al., 2018; Jiang et al., 2019; Botté and Potier, 2020; Filippone and Praticò, 2021). This connection between Down syndrome and AD could point toward endocytic processes as a potential driver of early endolysosomal dysfunction in AD.

Increased size and/or number of early endosomes is thought to correlate with an increase in general endocytosis, which results in the enlarged endosomal phenotype (Stenmark et al., 1994). This effect could indicate that changes in upstream endocytosis are important factors in downstream endolysosomal dysfunction in AD. The impact of cargo processed into this pathway has also been shown to alter endosomal function. For instance, in primary cortical rat neurons, internalization of Aβ_1-42_ resulted in increased levels and altered distribution of endolysosomal markers, as well as triggered neuronal degeneration. Inhibiting general endocytosis via phenylarsine oxide, and CME specifically with sucrose, attenuated this effect and returned endolysosomal markers to normal, suggesting that endocytosis of pathogenic Aβ_1-42_ contributes to downstream endosomal dysfunction (Song et al., 2011). Therefore, changes in CME may contribute to AD endolysosomal dysregulation in multiple ways.

Small et al. (2017) and Kimura and Yanagisawa (2018) summarize many of these endolysosomal disruptions into the “traffic jam” hypothesis. This hypothesis posits that disruptions in endocytosis and downstream endosome trafficking could be involved in altering the metabolism of AD related proteins and thus act as a pathogenic hub in AD. Similarly, Limone et al., proposed using the related genetic changes in endolysosomal trafficking as a “genetic hub” of AD which could contribute to many pathological events and illuminate potential drug targets (Limone et al., 2022). However, as these hypotheses focus primarily on trafficking of endosomes after endocytosis, how endocytosis itself may be altered and affect cellular phenotypes in the context of AD remains to be considered.

## 3 Clathrin mediated endocytosis in AD

CME has been well studied under normal physiological conditions, but little is known about how it may be changed during AD progression. In addition to its downstream connection with the endolysosomal pathway, several studies have indirectly implicated CME in AD in humans and animal models through genomics, transcriptomics, and proteomics of CME-related genes and proteins. A current summary of CME proteins associated with AD, their function in CME, and which AD brain regions are affected is presented in Table 1.

**Table 1:** CME proteins in human post-mortem AD brains.

Gene: protein name	Role in CME	Change in AD	Method	Brain region	References
*AP2A1:* AP-2 complex subunit alpha-1	Nucleation	Decreased	IHC	Layer II superior frontal gyrus	Yao et al. (2000)
		No change	IB	Total superior frontal gyrus, total hippocampus	
		Localized to NFTs	IHC	Temporal neocortex	Srinivasan et al. (2022)
*FCHO1:* FCH and mu domain containing endocytic adaptor 1	Nucleation	Unknown	–	–	–
*SNAP91:* Synaptosome associated protein a1 (AP180)	Paralog of PICALM, cargo selection	Decreased	IHC	All layers superior frontal gyrus, hippocampus CA3/Hilus	Yao et al. (1999)
		Decreased	IHC, IB	Dentate gyrus, CA3, entorhinal cortex, temporal cortex	Cao et al. (2010)
*ITSN1:* Intersectin 1	Nucleation, cargo selection	Increased	RT-PCR	Frontal cortex	Wilmot et al. (2008)
		no change	IB	Frontal cortex, temporal cortex	Malakooti et al. (2019)
*PICALM:* Phosphatidylinositol-binding clathrin assembly protein	Cargo selection	Increased	RT-PCR	Frontal cortex	Baig et al. (2010)
		Localized to NFTs	IHC	Hippocampus CA4	Ando et al. (2013), Alsaqati et al. (2023)
		Decreased	IB	Neocortex	
*CLTA*: Clathrin, light chain	Coat assembly, scission, uncoating	Localized to Some NFTs	IHC	Hippocampus	Nakamura et al. (1994)
*CLTC*: Clathrin, heavy chain		Increased	IHC	Frontal cortex	Alsaqati et al. (2023)
*DNM1:* Dynamin 1	Scission	Decreased	IHC	Hippocampus, entorhinal cortex	Cao et al. (2010)
*DMN2:* Dynamin 2	Scission	Decreased	qRT-PCR	Hippocampus	Aidaralieva et al. (2008)
		Decreased	RT-PCR	Temporal cortex	Kamagata et al. (2009)
*BIN1:* Amphiphysin 2	Scission	Increased	PCR	Frontal cortex	Chapuis et al. (2013)
		Decreased	IB	Frontal cortex	De Rossi et al. (2016)
*AMPH:* Amphyphysin 1	Paralog of BIN1, scission	Decreased	IB	Frontal cortex	De Jesus-Cortes et al. (2012)
*RAB5A*: Ras-related protein Rab-5A	Uncoating, early endosome fusion	Increased	IB	Frontal cortex	Alsaqati et al. (2023)
		Increased	qPCR, IB	Hippocampus CA1, frontal cortex	Ginsberg et al. (2010)

The table includes the gene and protein name, role in CME, change in protein in AD, method of detection, brain region evaluated, and reference(s). NFT, neurofibrillary tangles; IHC, Immunohistochemistry; IB, Immunoblot; PCR, Polymerase Chain Reaction; RT-PCR, Real-time PCR; qRT-PCR, Quantitative RT-PCR.

In 2018, Ahmad et al., calculated weighted genetic risk scores by combining data from the longitudinal AD Rotterdam Study (Hofman et al., 2015) and 20 AD genetic risk variants. These risk scores were then clustered within cellular pathways and associated with AD, mild cognitive impairment (MCI), and brain MRI phenotypes. The risk score of endocytic pathways was significantly associated with MCI, and the clathrin/AP-2 adaptor complex pathway was modestly associated with white matter lesions (Ahmad et al., 2018). Interestingly, other studies assessing genetic risk factors of AD found that the endocytosis pathway was significantly associated with reliance against AD (Tesi et al., 2020) and that abnormal Aβ levels, but not Tau, are significantly associated with endocytosis (Schork and Elman, 2023). These associations suggest an early involvement of endocytosis, specifically CME, in AD risk/etiology and potential resilience. Indeed, proteins involved in CME such as AP180 (cargo selection), AP-2 (cargo selection/clathrin recruitment), and Dynamin 1 (scission) are shown to be decreased in AD in various regions of human post-mortem brains at both the RNA and protein level (Yao et al., 1999, 2000, 2003; Cao et al., 2010; Piras et al., 2019). Together, the changes of CME protein levels point toward involvement of the pathway in AD and its potential to be both disease contributing or neuroprotective, but each protein must be evaluated individually to understand how its changes may affect or contribute to potential CME dysfunction in AD.

Several of these protein changes also translate to a transgenic mouse model of AD with a mutation in humanized *APP* (APP_SWE_) (Hsiao et al., 1996). Dynamin 1 and AP180 were decreased in APP_SWE_ brains versus controls, with varying degrees of specificity in subregions of the hippocampus, entorhinal cortex, and temporal cortex, similar to their changes in human AD brains (Cao et al., 2010). Interestingly, a separate study of the cortex in APP_SWE_ mice showed several CME proteins (APP, Clathrin, Dynamin 2, and PICALM) were upregulated, not downregulated as was observed in other brain areas. Importantly, proteins related to clathrin-independent endocytosis such as flotillin-1, and caveolin-1 and -3 were not changed (Thomas et al., 2011). These observations demonstrate that proteins specific to CME, and not to other common endocytic pathways, are changed and could point toward CME being specifically responsible for downstream endocytic abnormalities in APP_SWE_ mice. However, it is unknown whether more recently studied AD models with multiple transgenes, such as the 3xTg (Oddo et al., 2003) or 5xFAD lines (Oakley et al., 2006), recapitulate these changes or have CME disruptions. Several studies have shown that pharmacological modulation of downstream endolysosomal functions in 5xFAD mice (Cuddy et al., 2022) and in the 3xTg improved endolysosomal function and disease phenotypes (Kim et al., 2023), though how these manipulations may affect and be affected by upstream CME function is unclear.

Another consideration is whether normal aging affects levels of CME proteins and CME function. It is well known that the biggest risk factor of developing AD is aging, and a change in CME proteins over time could account for a possible connection between this risk factor and early cellular phenotypes. To date, only one study has attempted to address this possible connection. In 2018, Alsquati et al., examined how endocytosis-related proteins change in the frontal cortex of neurologically normal males from different age groups: 20–30 (young), 45–55 (middle aged), and 70–90 (old) (Alsaqati et al., 2018). Interestingly, both CME and clathrin-independent proteins were changed in an age-dependent manner. Four CME proteins, Dynamin I, isoform 4 of PICALM, AP180, and Rab5, were all significantly increased from the young to old age groups. Clathrin-independent proteins such as caveolin-2 and flotillin-2, increased with age, while caveolin-3 was decreased with age. Of note, some of these protein changes contrast with those seen in AD brains in the studies described above (Yao et al., 1999, 2000, 2003; Cao et al., 2010; Piras et al., 2019) and in Table 1, and may be related to general changes in endocytosis rather than specific changes to CME. It is unclear whether this effect may be due to evaluation of different brain regions, or to the exclusion of females from the aging study. Prior studies examining CME proteins in AD included both females and males in their cohorts, which is important, as females have a much higher incidence of AD (Rajan et al., 2021; Alzheimer's Association, 2023). Thus, examination of protein levels in males only does not allow any exploration of potential sex difference in endocytosis during aging, which could be a factor in the differential direction of expression changes between the aging study and others mentioned above. Additionally, the 15-year gap between each age group in these data should be considered. While the average age of AD diagnosis is about 75 years of age (Barnes et al., 2015), brain changes can occur for many years beforehand; thus, the 15 year gap between the middle aged (45–55) and old aged (70–90) groups could be enlightening and thus an important time point to evaluate. While the Alsquati study is a very promising initial study, more research is needed to elucidate the potential role of CME in early AD and if, or how, aging may be involved.

When thinking about how these observed differences in AD CME proteins may affect function, we must also consider the potential for specific CME functions in different brain regions and cell types. CME proteins show different changes depending on what brain region was analyzed (summarized in Table 1). Also, CME proteins may not change in the same way in all cells, since AD has varying effects on many brain cell types (e.g., neurons, astrocytes, and microglia) throughout disease progression (Maninger et al., 2024). Studies are still needed to examine how the effect on overall CME function might be different in each brain region depending on how a specific protein, or set of proteins, is changed. It is possible that an increase in one protein potentially reduces CME, while the same increase in a different protein could decrease CME in a different brain region. Additionally, depending on the cell type, dysregulation of CME proteins and resulting downstream dysfunction may have distinct cellular outcomes. However, studies using whole brain lysate are not able to distinguish protein expression in different cell types. Thus, studies focused on individual cell types are needed to establish whether CME is altered in distinct ways. Though brain region-specific changes are more challenging to evaluate due to difficulty obtaining the appropriate samples, many studies have begun to tease apart the potential cell type differences of CME proteins in AD, and will be further described in the remainder of this review.

## 4 CME dysfunction and AD cellular disruptions across multiple cell types

### 4.1 Neurons

#### 4.1.1 CME involvement in synaptic dysfunction

As AD is characterized by increasing loss of neurons throughout disease, many studies to date have focused on how altered neuronal functions may affect disease progression. It is well established that synaptic dysfunction, loss, and subsequent neuronal degeneration contributes to the progressive nature of AD. Reduced synapse numbers are observed throughout disease progression and are strongly correlated with cognitive decline (Terry et al., 1991), but synapses start to show dysfunction even before measurable cellular degeneration. Normally, functioning synapses rely on the quick release of pre-synaptic vesicles which are rapidly recycled via CME to maintain the pool of vesicles available for transmission (Soykan et al., 2016). Synaptic disruption in AD can be induced by both extracellular Aβ_1-42_ oligomers and intracellular hyperphosphorylated Tau, and as such, may be connected to other aspects of AD neuropathology, such as altered APP processing, abnormal phosphorylation, and calcium imbalance (Yao, 2004; Pei et al., 2020; Hori et al., 2022; Ratan et al., 2023). Of note, synaptic vesicle recycling is reduced in AD, and multiple CME proteins that are involved in maintenance of synaptic vesicle size, uniformity, and turnover, are clustered at pre-synaptic sites (Pechstein et al., 2010; Milosevic, 2018). While several other mechanisms work in parallel to CME to maintain the synaptic vesicle pool, studies have shown that blocking CME significantly reduces synaptic vesicle recycling (Soykan et al., 2016), indicating that CME is a major recycling mechanism, and supporting that disruption of this machinery could contribute to AD synapse dysfunction.

Synaptic dysfunction in AD can also result from disrupted regulation of AMPA receptors (AMPARs) in the post-synaptic cleft. Regulation of AMPARs at the membrane is involved in learning and memory, with receptor presentation leading to long-term potentiation (LTP) and internalization leading to long-term depression (LTD) (Chater and Goda, 2022). In AD, AMPAR trafficking is changed such that LTP is impaired and LTD is enhanced which contributes to cognitive deficits (Al-Hallaq et al., 2007; Pei et al., 2020; Babaei, 2021). In LTD, AMPARs are internalized via CME, and internalization can be induced by interaction with Aβ_1-42_ (Man et al., 2000; Lacor et al., 2004; Snyder et al., 2005; Guntupalli et al., 2017) suggesting that CME could be involved in the LTP/LTD changes in AD. Additionally, the AD risk factor and CME protein PICALM is involved in surface regulation of AMPARs. Loss of PICALM in mice initiates LTP and reduces LTD which improves learning (Azarnia Tehran et al., 2022). This directly implicates CME in AMPAR trafficking alterations and potential effects on learning and memory in AD. If CME dysfunction is an initial piece of this early synaptic dysfunction in multiple contexts, it could very well be an upstream driver in disruption of many neuronal functions.

#### 4.1.2 CME involvement in Aβ processing and uptake

As briefly mentioned above, in AD APP is preferentially processed into amyloidogenic Aβ_1-42_ in the endolysosomal pathway of neurons after internalization via CME (Koo and Squazzo, 1994; Wu and Yao, 2009). This processing occurs in the early endosome, where the enzyme BACE1 and APP are individually delivered, and APP is cleaved by BACE1 and α-secretase into the pathogenic Aβ_1-42_. Regulators of endosomal trafficking, which normally prevent BACE1 and APP from being delivered to the same compartment, have been identified as genetic risk factors for AD (*APOE4, PICALM, BIN1*) (Szabo et al., 2022; Maninger et al., 2024). Disrupted expression of these regulators could contribute to the increased production of Aβ_1-42_ in the endosome (Thomas et al., 2016; Guimas Almeida et al., 2018). After amyloidogenic cleavage of APP, Aβ_1-42_ peptides are secreted from the cell and aggregate to form toxic fibrils and plaques (Zhang et al., 2011). In aged mouse hippocampal neurons, APP endocytosis is reduced with CME inhibition, but it is unclear if this directly results in less Aβ_1-42_ production (Burrinha et al., 2021). How CME alterations directly affect APP internalization and Aβ_1-42_ production in an AD model has not yet been closely studied, but it is plausible that increased CME could cause a subsequent increase in APP internalization and processing, and conversely, that decreased CME may therefore decrease Aβ_1-42_ production.

The production of Aβ_1-42_ has also been connected to synaptic activity, which can stimulate CME and increase the internalization of APP. In fact, electrical stimulation in the hippocampus of the APP_swe_ mouse model increased Aβ_1-42_ in brain interstitial fluid but was prevented up to 70% by CME inhibition using a Dynamin dominant-negative inhibitory peptide (Cirrito et al., 2005, 2008). This result suggests that endocytosis, both synaptic activity-mediated and clathrin-mediated, affects the levels of Aβ_1-42_ produced and released from neurons, which could contribute to the development of extracellular Aβ_1-42_ aggregation and spreading.

In addition to APP being endocytosed through CME, different forms of processed Aβ have also been shown to be internalized into neurons and accumulate in insoluble aggregates within endolysosomal compartments in AD brains (Gouras et al., 2000). This accumulation of Aβ_1-42_ in late endosomes and lysosomes could contribute to endolysosomal dysfunction and associated cellular disruptions (Colacurcio et al., 2018; Lai et al., 2021). Furthermore, endocytosed Aβ_1-42_ can also cause intraneuronal dysfunction outside of lysosomal accumulation; for example, cultured rat hippocampal neurons treated with Aβ_1-42_ showed decreased synaptic vesicle endocytosis and Dynamin 1 expression after stimulation, indicating that the replenishing of vesicles to the readily available pool is impaired by Aβ_1-42_ (Cao et al., 2010). Another of many observed intercellular effects of Aβ_1-42_ fibrils is the induction of neuritic defects and growth cone collapse. In cultured cortical neurons, Aβ_1-42_ internalization can result in failure of axonal repair and contribute to axonal degeneration (Kuboyama et al., 2005; Laferla et al., 2007). It has since been shown that endocytosis in growth cones is increased after Aβ_1-42_, and that inhibition of CME prevents the cone collapse (Kuboyama et al., 2015) suggesting that CME is a primary mechanism driving downstream effects of Aβ_1-42_.

However, there are conflicting reports as to which endocytic mechanism is responsible for taking up each form of processed Aβ (e.g., monomeric, oligomeric, or fibrillar Aβ_1-42_). One study that evaluated the uptake of monomeric non-pathogenic Aβ_1-40_ and pathogenic Aβ_1-42_ in SH-Sy5Y neuroblastoma cells found that uptake of Aβ_1-42_ is twice as efficient as that of Aβ_1-40_ and is primarily independent of CME (Wesén et al., 2017). A subsequent study from the same group found that while the mechanism of Aβ uptake observed was similar to some established endocytic pathways, there are likely more undiscovered molecular components regulating Aβ_1-42_ uptake (Wesén et al., 2020). Yet other studies report that several forms of Aβ are internalized through CME in different cell types. For example, in rat primary hippocampal neurons, prefibrillar oligomeric forms of Aβ_1-42_ were rapidly taken into cells in a Dynamin-dependent manner, accumulated in lysosomes, and caused disruption to endolysosomal function. Importantly, a non-misfolded variant of Aβ_1-42_ was not internalized and did not show any effects on endolysosomal function (Marshall et al., 2020). In addition, in mouse neuro-2a cells, both Aβ_1-42_ monomers and oligomers showed minimal capacity for binding with the membrane itself but were not able to enter cells under CME inhibition (Shi et al., 2020), meaning that toxicity of Aβ_1-42_ fragments could be due to their internalization through receptors.

These observed differences in uptake methods for each Aβ_1-42_ form could be due to the use of multiple experimental models or structural changes in receptor binding to initiate endocytosis. They point to aggregation state of Aβ_1-42_ as an important factor to consider when evaluating the mechanisms and further effects of Aβ_1-42_ internalization. Indeed, several studies have shown that oligomeric and aggregated Aβ_1-42_ are internalized more and are more toxic to neurons than fibrillar or monomeric Aβ_1-42_ (Chafekar et al., 2008; Jin et al., 2016). However, these studies do not clarify which mechanism of endocytosis is involved, clathrin mediated or otherwise. Additionally, when comparing uptake of pathogenic Aβ_1-42_ vs. non-pathogenic Aβ_1-40_, Aβ_1-42_ and not Aβ_1-40_ is internalized by Dynamin-dependent endocytosis (Omtri et al., 2012) which can include more endocytic processes than purely CME (Mayor et al., 2014; Rennick et al., 2021). Taken together, it is still unclear how each different form of Aβ is internalized into neurons and what downstream effects they may have inside the cell. Nevertheless, there are many indications that CME is clearly an important player in neuronal AD mechanisms and should be studied further to tease the emerging nuances apart.

#### 4.1.3 CME involvement in Tau dysfunction

While we have thus far focused on how CME disruptions may relate to Aβ pathology, it may also play a role in Tau dysfunction. During its propagation, pathological Tau is excreted and then internalized by neighboring cells (Mohamed et al., 2013). Internalization of extracellular Tau aggregates, as opposed to monomers, has recently been suggested to initiate Tau propagation in recipient cells (Usenovic et al., 2015; Kolarova et al., 2017). Uptake of Tau has been observed to occur through many types of endocytosis, including phagocytosis, Dynamin-dependent endocytosis, bulk endocytosis, CME, and others (Calafate et al., 2016; Ando et al., 2020; De La-Rocque et al., 2021; Zhao et al., 2021). However, as with CME effects on Aβ, there are few direct experiments that examine how CME disruption in AD may affect Tau propagation and resulting neuronal dysfunction or vice versa.

In non-disease state, it has been shown that heathy human neurons uptake both aggregated and soluble Tau in similar ways (Evans et al., 2018) and so Tau internalization may not be solely a toxic disease process. On the other hand, cultured mouse neurons with overexpressed human Tau show pre-synaptic endocytosis deficits by decreasing Dynamin RNA and protein but not clathrin, indicating that dysregulation of Tau, such that occurs in AD, may interfere with synaptic transmission via endocytic disruption (Xie et al., 2019). Additionally, a fragment of BIN1, a CME protein and AD risk factor, accelerates Tau aggregate uptake in primary cultured neurons. This process is inhibited by Dynasore, a Dynamin-dependent endocytosis inhibitor which reduces CME and several other Dynamin-dependent endocytic processes (Mayor et al., 2014; Zhang et al., 2024). Of note, it has been reported that inhibition of clathrin specifically does not reduce Tau endocytosis (Holmes et al., 2013; Wu et al., 2013), so these effects could be mediated by non-CME specific processes that still rely on Dynamin.

It is also important to consider that it may be difficult to distinguish AD-specific Tau endocytic changes from changes in other tauopathies. One study applied brain derived Tau oligomers from human post-mortem tissue of tauopathies including AD, progressive supranuclear palsy, and dementia with Lewy bodies to mouse primary neurons and showed that Tau oligomers from each disease state were internalized via the same non-Dynamin dependent endocytic mechanisms (Puangmalai et al., 2020). Thus, as multiple endocytic pathways seem to be involved, more work needs to be done to tease apart the nuances of how CME specifically might affect or be affected by Tau pathology in healthy neurons and in AD versus other tauopathies.

### 4.2 Astrocytes

As CME is a ubiquitous process, changes in non-neuronal cell types during AD must be considered. Here, we will focus on astrocytes and microglia, which have myriad functions essential for maintaining a healthy nervous system and respond quickly to changes in environment (Vainchtein and Molofsky, 2020). Both have been implicated in reactivity to and protection from AD pathology, often in synchrony (Al-Ghraiybah et al., 2022).

Astrocytes perform many essential functions in a healthy central nervous system, including regulating synapse activity, interactions with blood vessels and endothelial cells to facilitate the blood brain barrier, neurogenesis, and maintenance of neuronal homeostasis. Astrocytes have diverse morphology and mechanisms of action for these functions (Eroglu and Barres, 2010; Sofroniew and Vinters, 2010; Sofroniew, 2020; Torres-Ceja and Olsen, 2022). In AD, many astrocytes become reactive, localize around Aβ_1-42_ plaques, and excrete inflammatory cytokines that contribute to widespread neuroinflammation and subsequent neuronal dysfunction (Al-Ghraiybah et al., 2022). It has also been shown that activated microglia can induce astrocytes to lose neuronal support abilities, and these neurotoxic astrocytes are abundant in AD and other neurodegenerative diseases (Liddelow et al., 2017).

Astrocytes are closely tied to the clearance of dying neurons and take up large amounts of neuronal Aβ_1-42_ and debris (Yuan et al., 2023; Mun et al., 2024). However, astrocytes can become overburdened with Aβ, resulting in impaired phagocytosis of diseased synapses and potentially contributing to the increased amount of dystrophic neurites seen in early stages of AD (Sanchez-Mico et al., 2021). Additionally, Aβ protofibrils engulfed by mouse primary astrocytes are not degraded in the cell and induce lysosomal dysfunction within them (Söllvander et al., 2016). Astrocytes of aged primate brains show enlarged early endosomes filled with Aβ, suggesting that aging may also play a role in astrocytic dysfunction (Kimura et al., 2014). And while it is largely thought that astrocytes internalize Aβ through phagocytosis, it has recently been shown that multiple forms of Aβ (1-40 and 1-42) are internalized via clathrin-coated vesicles in rat primary astrocytes, where they induce increased reactive oxygen species production and decreased cell viability. When treated with chlorpromazine, a pharmacological inhibitor of CME, astrocytes exhibit significantly decreased uptake of Aβ_1-42_ and a partial rescue of Aβ_1-42_ induced cell death (Domínguez-Prieto et al., 2018). Additionally, astrocytes can take up and accumulate monomeric and oligomeric Tau (Martini-Stoica et al., 2018; Brezovakova et al., 2022; Eltom et al., 2024; Giusti et al., 2024), though to the best of our knowledge the specific endocytic mechanisms involved have not been thoroughly examined and there is no strong link to CME specifically in astrocytes.

Another consideration of astrocytic involvement in AD is their connection to the genetic risk factor of apolipoprotein E4 (*APOE4*). *APOE* is mainly expressed in astrocytes as their primary cholesterol carrier, and expressing the *APOE4* allele is one of the biggest risk factors for developing AD (Martens et al., 2022). Many AD phenotypes are affected by *APOE* status including Aβ clearance and lipid metabolism (Koistinaho et al., 2004; Kim et al., 2009; Lin et al., 2018). Human iPSC derived astrocytes with *APOE4* exhibit both decreased CME and early endosomal markers, suggesting that *APOE4* disrupts CME. Interestingly, increased expression of *PICALM* rescues endocytic defects in the *APOE4* astrocytes (Narayan et al., 2020). The connection of these two major genetic risk factors of AD through CME suggests that CME is instrumental in disease development.

Taken together, it is possible that the changes in CME protein levels observed in AD post-mortem brains contribute to the dysfunction of astrocytes and their role in driving subsequent AD pathology. Moreover, the involvement of CME in astrocytic responses to Aβ_1-42_ illustrates the importance of evaluating CME in cell types other than neurons. It also opens questions regarding how aberrant CME early in AD contributes to non-neuronal disease phenotypes.

### 4.3 Microglia

Microglia, central nervous system resident phagocytes, are well recognized to have a dual role in AD, as reviewed recently (Cai et al., 2014; Al-Ghraiybah et al., 2022; Wendimu and Hooks, 2022; Zhao et al., 2022). Briefly, microglia can act in both a neurotoxic and neuroprotective role in AD, and which role they perform depends on many factors. Microglia activated from their resting state into neurotoxic phenotypes promote neuroinflammation through release of neurotoxic cytokines and other inflammatory factors. Contrasting this response, activated microglia in their neuroprotective phenotype can limit plaque formation by clearing extracellular Aβ_1-42_. Paradoxically, both phenotypes can be initiated by Aβ_1-42_. One prominent hypothesis in AD pathogenesis is that an initial problem that arises in disease progression is the loss of neuroprotective microglial function due to age-associated microglial senescence, which in turn leads to loss of Aβ_1-42_ clearance and accumulation of Aβ_1-42_. This accumulation of Aβ_1-42_ then activates microglial neurotoxic states, which increase neuroinflammation and eventually leads to dementia (Streit et al., 2021). This idea implicates microglia, and specifically a disruption in Aβ_1-42_ clearance, in early stages of AD.

Endocytic clearance of Aβ_1-42_ by microglia can occur through several mechanisms, the most commonly studied being micropinocytosis and phagocytosis (Ni et al., 2024). However, recent studies have begun to implicate CME in this process. In HMO6 cells, a human microglial cell line, treated with an amyloid-degrading enzyme activator, Aβ_1-42_ uptake was significantly increased, and both clathrin and caveolin expression were upregulated (Jang et al., 2015). However, no studies were done to examine the effect of inhibiting clathrin- or caveolae-dependent endocytosis independently, so it is unclear whether one process is more prominent that the other. Nevertheless, these findings suggest an involvement of CME in microglial Aβ_1-42_ clearance, although it may not be the only mechanism at play.

More recently, a study using a mouse microglial cell line treated with fibrillar Aβ_1-42_ found that clathrin colocalized with internalized Aβ_42_ and that inhibition of CME resulted in 80% reduction of Aβ_1-42_ uptake (Fujikura et al., 2019), further supporting the hypothesis that CME is involved in microglial Aβ uptake. Additionally, in another mouse microglia cell line, a novel form of endocytosis has been described, autophagy protein Light Chain 3 (LC3)-associated endocytosis (LANDO) (Heckmann et al., 2019). LANDO supports the clearance of Aβ and prevents activation of inflammatory microglia, yet inhibition of actin polymerization and phagocytosis had no effect on internalization of Aβ. This finding suggests that LANDO occurs though CME, as LC3 is colocalized with clathrin and Rab5 early endosomes (Heckmann et al., 2019). Also, it is important to note that these studies have not yet been replicated *in vivo* and should be validated further, as microglia function is strongly influenced by the surrounding environment. However, the recent evidence of CME in microglial clearance of Aβ_1-42_ does point toward another potential effect of CME disruption in AD. When considering microglial involvement in Tau pathology, to our knowledge there has been no specific link between microglial uptake and propagation of Tau via CME mechanisms. How microglia contribute to Tau pathology is reviewed in depth elsewhere (Das et al., 2020; Ayyubova, 2023; Chen and Yu, 2023; Ou-Yang et al., 2023). CME in microglia needs to be further investigated to clarify what degree it and other endocytic mechanisms are disrupted in AD in relation to both Aβ and Tau pathology. Reduction in CME could contribute to neurotoxic microglial function through reduction of Aβ_1-42_ clearance. Alternatively, if CME is not changed or is increased in AD microglia, it could be driving neuroprotection of microglia, whereby Aβ_1-42_ clearance is actually contributing to a slowing of disease progression.

## 5 Targeting CME for AD treatment

Currently, most clinical treatments for AD are Aβ-targeting monoclonal antibodies and have varying success in reducing Aβ burden and slowing cognitive decline (Alshamrani, 2023; Rabinovici and La Joie, 2023). However, they are also most effective in early stages of disease, as characterized by Aβ and Tau levels (Alshamrani, 2023). Aβ burden can be decreased in some patients, but progression is not completely halted (Sevigny et al., 2016; Mintun et al., 2021; Swanson et al., 2021; Budd Haeberlein et al., 2022) suggesting that targeting Aβ is not sufficient to significantly alter other molecular pathways involved in later stage AD pathophysiology. Therapies targeting Tau have recently become more heavily studied with several immunotherapies in clinical trials (Ji and Sigurdsson, 2021; Alshamrani, 2023). However, early intervention is still sorely needed for patients and modulation of other AD pathophysiology outside of targeting Aβ and Tau directly are being considered for treatment (Zhang et al., 2021).

As discussed within this review, there is abundant evidence that CME is a potential early disease modifier of AD in neurons, astrocytes, and microglia upstream of endolysosomal involvement. There have been several clinical trials evaluating endocytosis inhibitors which specifically block CME to treat coronavirus infection (Szewczyk-Roszczenko et al., 2023). These inhibitors include Ruxolitinib and Simvastatin, Chlorpromazine, and most interestingly Hydroxychloroquine (HCQ) which reduces PICALM expression (Szewczyk-Roszczenko et al., 2023). HCQ is a common treatment for arthritis and in 2001 was found to have no significant benefit to early AD (Van Gool et al., 2001). More recently, HCQ was found to reduce dementia risk in humans and rescued AD phenotypes *in vitro* and *in vivo* (Varma et al., 2023). While HCQ treatment is promising, its mechanism of action in specific cell types should be evaluated as it can also affect other non-CME pathways (Schrezenmeier and Dörner, 2020). Thus far, to our knowledge no other endocytic inhibitors have been applied to a clinical context of AD.

So, while there is great therapeutic potential of modulating CME in AD due to the many ways its disruption can interface with aspects of disease pathophysiology, it is also clear that treatments must be targeted to specific cell types. This is an area of interest for the delivery of anticancer drugs to specifically target diseased tissue and cells and reduce off target effects (Zhao et al., 2020; Mitchell et al., 2021) the application of which could be applied to AD treatment in the future. Additionally, since CME itself can be a mechanism of uptake for therapeutics (Congdon et al., 2013; Rennick et al., 2021), if its function is disrupted in AD already, drug delivery into cells may be compromised and should also be considered. There is still much to unravel before potential therapeutic developments using CME modulation for AD are possible, such as the directionality of CME functional changes and how it may vary between cell types.

## 6 Conclusion

In conclusion, CME is emerging as a critical player in the development and progression of AD in multiple brain cell types. CME is an important intersection between early neuronal dysfunction via Aβ_1-42_ accumulation and downstream effects that contribute to neuronal viability, such as synaptic vesicle recycling deficits and disruption of AMPAR regulation. Not only is Aβ_1-42_ produced following CME of APP, but internalization of multiple forms of extracellular Aβ_1-42_ contribute to downstream neuronal pathology. In astrocytes and microglia, which are heavily involved in clearance of extracellular Aβ_1-42_ in the brain, CME disruptions reduce internalization of Aβ_1-42_. Importantly, other brain cell types, including endothelial cells, oligodendrocytes, and ependymal cells that are not reviewed herein, should also be considered in the context of CME and AD as they could also be affected by changes in CME. Further, other copathologies associated with AD that may also affect or be affected by CME disruption, such as TDP-43 (Root et al., 2021), should be considered. Ultimately, CME appears to be involved in dysfunction in multiple cell types across early and late stages of AD. Thus, elucidating the mechanisms of CME and how their disruption is related to AD pathogenesis or neuroprotection in each brain cell type would both lead to a better understanding of AD mechanisms and potentially point to novel targets for the treatment or prevention of AD.

## Author contributions

SJ: Writing – review & editing, Conceptualization, Data curation, Investigation, Writing – original draft, Funding acquisition. US: Supervision, Writing – review & editing. JS: Supervision, Writing – review & editing.
